# Application of multiscale magnetotelluric data to mineral exploration: an example from the east Tennant region, Northern Australia

**DOI:** 10.1093/gji/ggac029

**Published:** 2022-02-16

**Authors:** Wenping Jiang, Jingming Duan, Michael Doublier, Andrew Clark, Anthony Schofield, Ross C Brodie, James Goodwin

**Affiliations:** Geoscience Australia, Canberra, Australian Capital Territory, Geoscience Australia, Canberra, 2600, Australia; Geoscience Australia, Canberra, Australian Capital Territory, Geoscience Australia, Canberra, 2600, Australia; Geoscience Australia, Canberra, Australian Capital Territory, Geoscience Australia, Canberra, 2600, Australia; Centre for Exploration Targeting, School of Earth and Environment, The University of Western Australia, 35 Stirling Highway, Crawley 6009, WA, Australia; Geoscience Australia, Canberra, Australian Capital Territory, Geoscience Australia, Canberra, 2600, Australia; Geoscience Australia, Canberra, Australian Capital Territory, Geoscience Australia, Canberra, 2600, Australia; Geoscience Australia, Canberra, Australian Capital Territory, Geoscience Australia, Canberra, 2600, Australia; Geoscience Australia, Canberra, Australian Capital Territory, Geoscience Australia, Canberra, 2600, Australia

**Keywords:** Electrical properties, Magnetotellurics, Inverse theory, Crustal structure

## Abstract

The footprint of a mineral system is potentially detectable at a range of scales and lithospheric depths, reflecting the size and distribution of its components. Magnetotellurics is one of a few techniques that can provide multiscale data sets to image and understand mineral systems. We have used long-period data from the Australian Lithospheric Architecture Magnetotelluric Project (AusLAMP) as a first-order reconnaissance survey to resolve large-scale lithospheric architecture for mapping areas of mineral potential in northern Australia. The 3-D resistivity model reveals a broad conductivity anomaly extending from the Tennant Creek district to the Murphy Province in the lower crust and upper mantle, representing a potential fertile source region for mineral systems. Results from a higher-resolution infill magnetotelluric survey reveal two prominent conductors in an otherwise resistive host whose combined responses result in the lithospheric-scale conductivity anomaly mapped in the AusLAMP model. Integration of the conductivity structure with deep seismic reflection data reveals a favourable crustal architecture linking the lower, fertile source regions with potential depositional sites in the upper crust. The enhanced conductivity likely resulted from the remnant (metallic) material deposited when fluids were present during the ‘ancient’ tectonic events. This observation strongly suggests that the deep-penetrating major faults potentially acted as pathways for transporting metalliferous fluids to the upper crust where they could form mineral deposits. This result and its integration with other geophysical and geochronological data sets suggest high prospectivity for major mineral deposits in the vicinity of these major faults, that is, Gulunguru Fault and Lamb Fault. In addition to these insights, interpretation of high-frequency magnetotelluric data acquired during the infill survey helps to characterize cover and assist with selecting targets for stratigraphic drilling which, in turn, can validate the models and improve our understanding of basement geology, cover sequences and mineral potential. This study demonstrates that integration of geophysical data from multiscale surveys is an effective approach to scale reduction during mineral exploration in covered terranes with limited geological knowledge.

## 1 INTRODUCTION

Approximately 80 per cent of Australia is covered by sedimentary basins and regolith, and as a consequence is largely underexplored. To discover new provinces for mineral exploration, Geoscience Australia has undertaken a series of integrated studies to identify prospective regions using new geological, geophysical and geochemical data collected under the Exploring for the Future (EFTF) program, together with legacy data sets. The underpinning framework for these activities is the mineral systems concept that distinguishes four main components essential for mineral deposit formation (Wyborn *et al*. 1994; Skirrow *et al*. 2019): (1) sources or ore metals, sulphur, fluids and ligands; (2) energy drivers that facilitate fluid flow and transfer of mass and energy; (3) favourable architecture to focus flux of metals, fluids and energy and (4) deposition mechanisms representing physico-chemical gradients that facilitate mineral precipitation. If the four components coincide in time and space, material from a source is transported to a deposition site via architecture connections to form a mineral deposit. Within this conceptual framework, the footprint of a mineral system is potentially detectable at a variety of scales, mirroring the range of scales of the main components, from ore deposits to Earth's lithosphere.

Electrical conductivity of earth materials varies due to rock composition, temperature and geochemical constituents including metallic sulphides, graphite, iron oxide and fluids (Ferguson *et al*. 1999). As a result, magnetotellurics (MT) is one of a few techniques that can provide multiscale data sets to map the footprint of mineral systems. It has been successfully used to detect key mineral system components at a range of scales, in conjunction with other geoscientific data sets (Heinson *et al*. 2006; Skirrow *et al*. 2018; Schofield *et al*. 2020).

**Figure 1.**
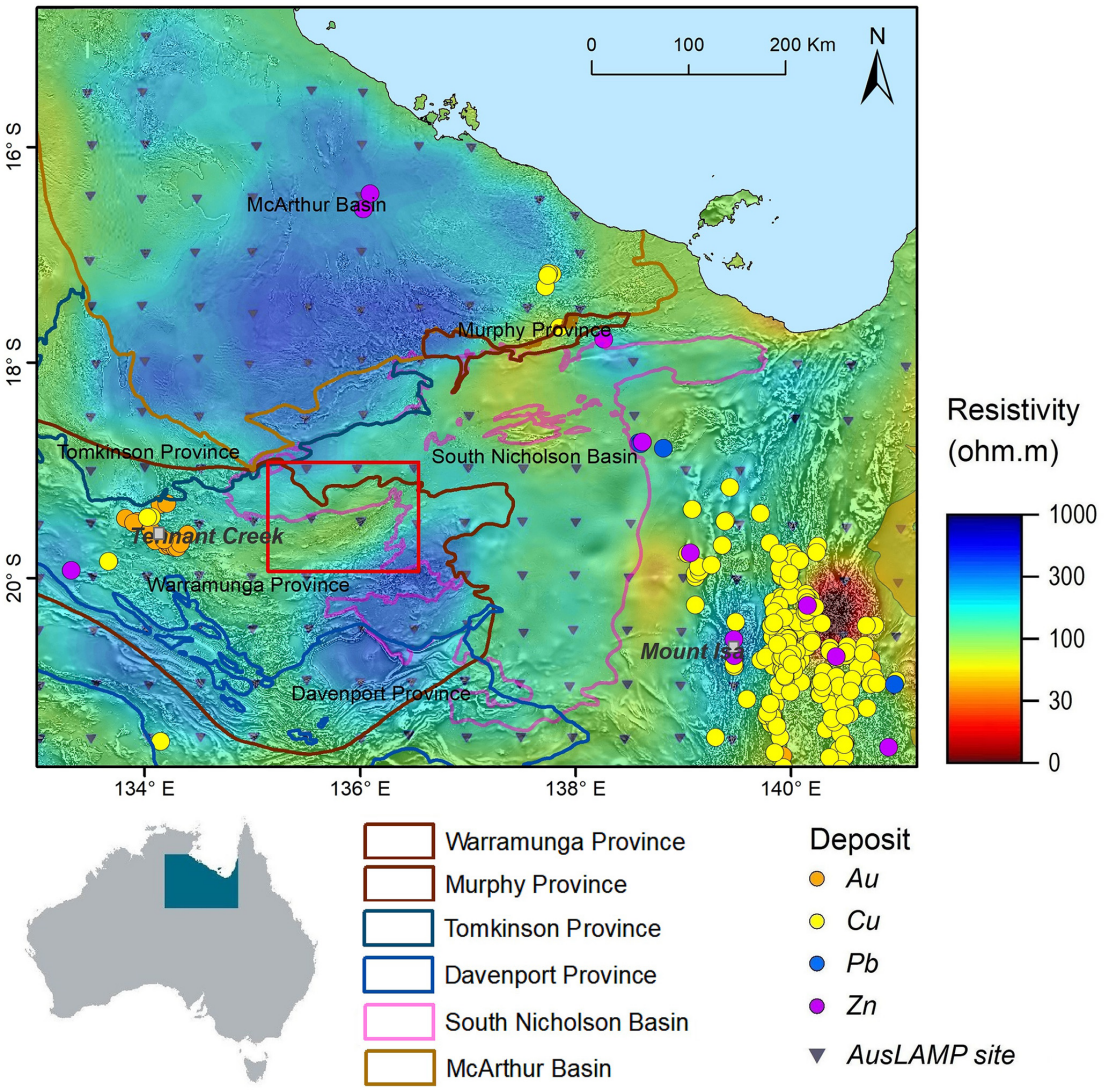
3-D resistivity model at a depth of ∼35 km derived from the EFTF program AusLAMP data set (Duan 2019; Duan *et al*. 2020) overlain on the total magnetic intensity anomaly map in grey scale (Nakamura & Milligan 2015). The red box shows the location of the infill MT survey in the East Tennant region.

A systematic assessment of mineral prospectivity often involves mapping and identification of large-scale footprints of a deposit-forming system, normally expressed deep in Earth's crust and mantle. This is particularly important for greenfields exploration decisions, that is, in regions where mineral deposits are not already known to exist, at regional, district or even craton scales. The Australian Lithospheric Architecture Magnetotelluric Project (AusLAMP) acquires long-period MT data on a half-degree grid spacing (∼55 km) across the entire Australian continent with the aim of deciphering the first-order conductivity structure of the continent. As part of the EFTF program, Geoscience Australia has significantly progressed AusLAMP data coverage throughout the Northern Territory and western Queensland, including many regions covered by (stacked) sedimentary basins. Resistivity models derived from the newly-acquired AusLAMP data (Fig. 1) have mapped large-scale conductivity anomalies not only in known highly endowed mineral provinces, but also in greenfield areas where mineralization has not been previously recognized (Duan 2019; Duan *et al*. 2020). For example, the mapped Carpentaria Conductivity Anomaly east of Mount Isa shows a close spatial correlation with the Cloncurry iron oxide copper-gold (IOCG) district, Queensland (Wang *et al*. 2018; Jiang *et al*. 2019; Duan *et al*. 2020). Similar conductive features are mapped in the Tennant Creek district in northern Australia where IOCG-type deposits have been discovered in exposed Palaeoproterozoic rocks (Skirrow *et al*. 2019). In addition, the AusLAMP model (Duan 2019; Duan *et al*. 2020) reveals a conductivity anomaly to the east of the largely exposed Tennant Creek district, representing a potential fertile source region for mineral systems. This conductivity feature coincides with a broadly east-northeast-trending corridor of shallowly, but completely, covered basement that is marked by a series of large-scale structures identified from potential field data and deep seismic reflection data (Clark *et al*. 2021; Southby *et al*. 2021). This underexplored region, referred to as the East Tennant basement high, is considered to have significant mineral potential (Skirrow *et al*. 2019; Czarnota *et al*. 2020; Murr *et al*. 2020; Schofield *et al*. 2020).

**Figure 2.**
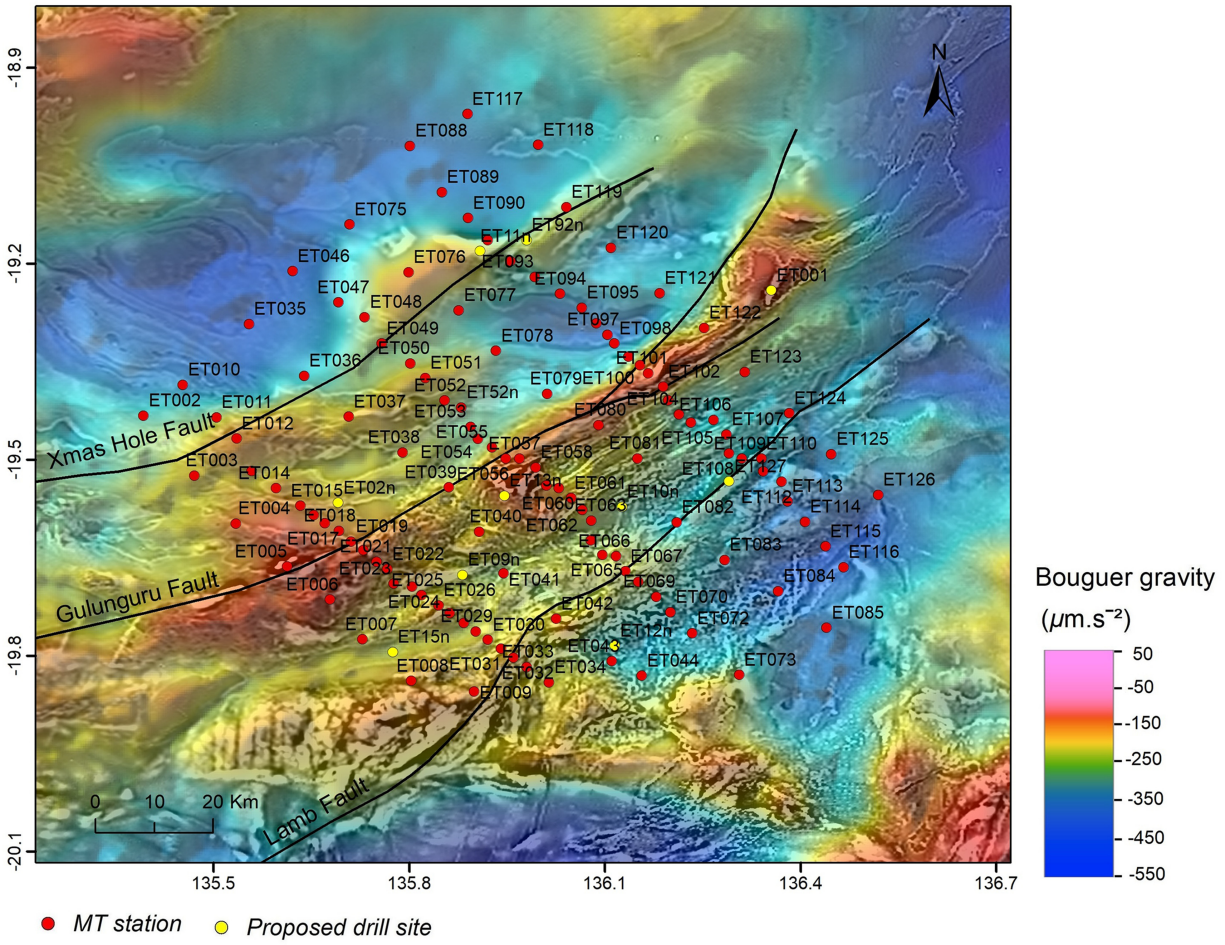
Infill broad-band and audio-MT stations in the East Tennant region. Black lines show the trace of major faults preliminarily interpreted from seismic reflection and potential field data (Clark *et al*. 2021). The background map is the Bouguer gravity anomaly map that combines the new gravity data in the East Tennant region (Wynne 2019) with the national gravity data (Nakamura 2016) overlain on the total magnetic intensity anomaly map of Australia, 6th Edition (Nakamura & Milligan 2015).

Based on this initial assessment, infill surveys were conducted to acquire higher-resolution gravity and MT data in the East Tennant region to better constrain the geological architecture and to improve understanding of mineral potential. In particular, we used broad-band MT (BBMT) data from the infill survey (Fig. 2) to image crustal architecture and to characterize the geometry of major structures. A key question addressed by the infill survey was whether the deep conductivity anomaly detected by the AusLAMP data is connected to features in the near-surface, potentially via the structural architecture. We also utilize deep seismic reflection data that transects the north-eastern part of the East Tennant region, to provide further constraints on the structural architecture of the area. Moving to the near-surface, we have used audio MT (AMT) data to constrain cover thickness for the selection of drilling targets for a stratigraphic drilling program which, in turn, will test models and further improve our understanding of basement geology, cover sequences and mineral potential.

In this paper, we present 3-D data inversion of the infill MT data for mapping crustal architecture, using a deterministic modelling approach. We also present a 1-D probabilistic inversion of the AMT data for cover thickness estimation, using a newly developed trans-dimensional Markov chain Monte Carlo algorithm. Preliminary interpretation of the resistivity models is supported by deep seismic reflection data, and integration with other geophysical and geochronological data sets within a mineral system framework sheds lights on mineral systems prospectivity in the East Tennant region.

## 2 GEOLOGICAL SETTING

**Figure 3.**
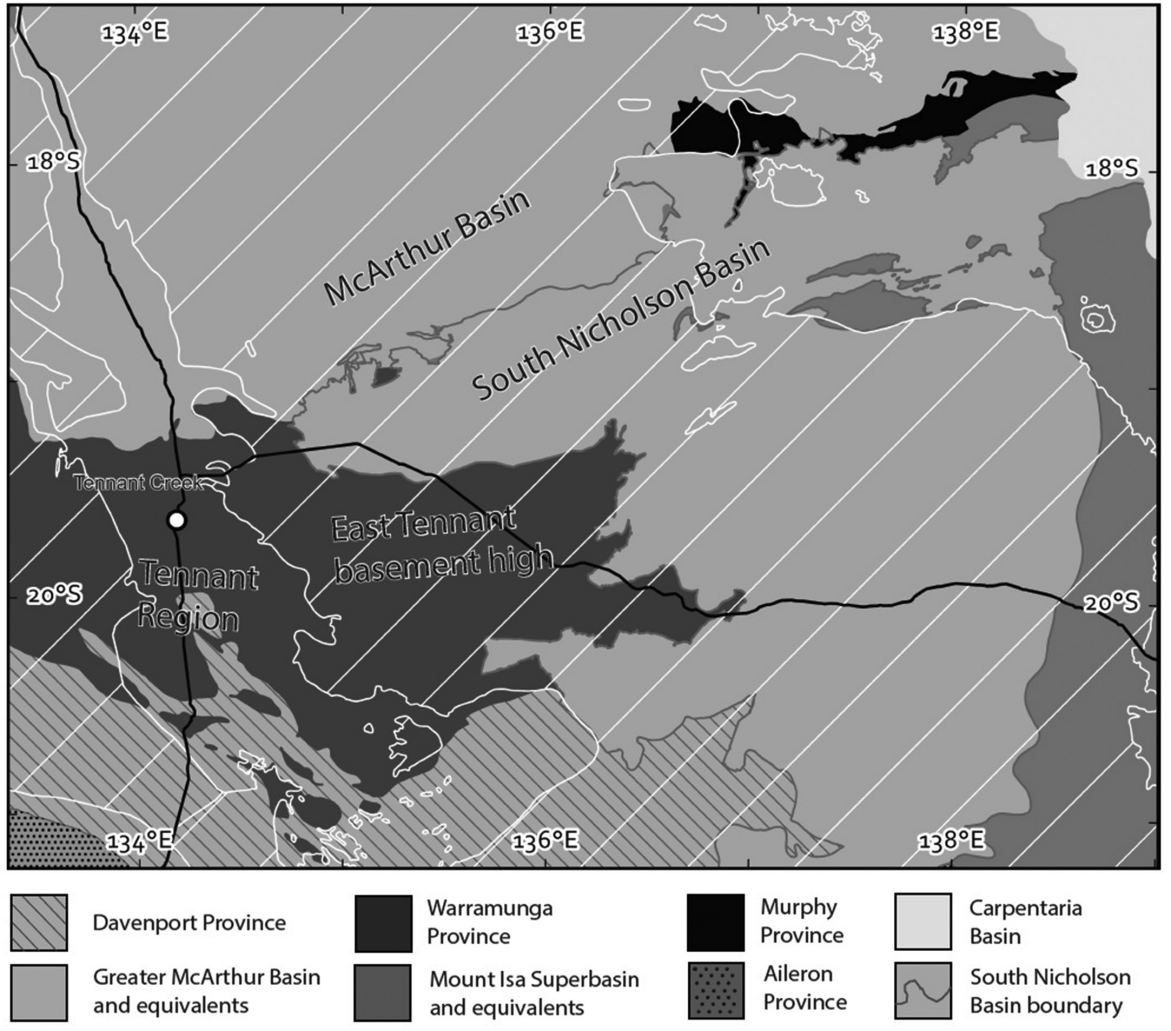
Simplified geological map showing Paleo-Mesoproterozoic orogens and basins of the Tennant Region, and neighbouring McArthur Basin and Mount Isa Province. The map is produced using Australian Geological Provinces 2018.01 edition (Raymond *et al*. 2018). The pre-1800 Ma orogens of the Tennant Region and East Tennant basement high are part of the Warramunga Province; the white hatched pattern delineates the Georgina and Wiso basins.

This study is focused on the central portion of a recently identified tectono-magmatic belt that stretches from the Tennant Region in the southwest to the Murphy Province in the northeast (Fig. 3, Cross *et al*. 2020; Schofield *et al*. 2020; Clark *et al*. 2021). It is concealed by relatively shallow cover rocks, and therefore, is referred to as the East Tennant basement high (Czarnota *et al*. 2019). This belt is characterized by a pre-1850 Ma deep-water stratigraphic package that was tightly folded, sheared, variably metamorphosed, and intruded by voluminous felsic magma at ∼1850 Ma, immediately prior to being overlain by felsic volcanics after ∼1850 Ma (Donnellan 2013; Cross *et al*. 2020). In the Tennant Region and the East Tennant basement high, these rocks belong to the Warramunga Province, whereas in the northeast they are collectively known as the Murphy Province (Fig. 3).

The age and nature of basement beneath the Warramunga Province is unknown, however, isotopic signatures from widespread intrusive rocks suggest that the Province is underlain by Archean cratonic basement (Wyborn *et al*. 1987; Champion 2013). The oldest rocks in the Warramunga Province consist of a widespread package of turbiditic metasedimentary rocks, with minor volcanics and carbonates, deposited at around 1860 Ma (Donnellan 2013; Cross *et al*. 2020). This package underwent significant deformation and sub-greenschist to amphibolite-facies metamorphism between ∼1855 and 1845 Ma, broadly coeval with an episode of voluminous felsic magmatism. This tectono-magmatism collectively defines the Tennant Event in the Warramunga Province (Donnellan 2013). Potential-field geophysical imagery, deep-crustal seismic and observations from drill-core reveal that regional-scale, south-dipping faults and shear zones that developed during the Tennant Event extend east from the Tennant Region into the East Tennant basement high and continue to the northeast towards the Murphy Province (Clark *et al*. 2021). Although the Warramunga Province experienced multiple episodes of deformation after the Tennant Event, these latter episodes are characterized by brittle reactivation of older shear zones and foliation, and gentle to open folding (Donnellan 2013, and references therein). Therefore, the main structures mapped in the Warramunga Province mostly reflect a tectonic architecture established between 1855 and 1845 Ma (Donnellan 2013; Cross *et al*. 2020; Clark *et al*. 2021).

Outcrop of the Warramunga Province is restricted to the Tennant Region (Fig. 3). Less-deformed Precambrian strata of the Greater McArthur Basin and Davenport Province unconformably overlie the Warramunga Province to the North and South, respectively, and rapidly thicken away from basement outcrop. However, immediately east of the Tennant Region Precambrian strata are largely absent, resulting in relatively thin cover depths of around 100–200 m that characterize the East Tennant basement high (Fig. 3). The East Tennant basement high is bounded to the north, east and south by thick sediments of the South Nicholson Basin, which is the age equivalent of the upper package of the Greater McArthur Basin (Hollis *et al*. 2010; Ahmad *et al*. 2013). It is worth noting that the South Nicholson Basin is known to contain locally thick sequences of organic-rich shales, such as the Mullera Formation, which are likely to be imaged by geophysical methods that measure conductivity (Carter & Zimmerman 1960). Both the South Nicholson Basin and the East Tennant basement high are covered by the relatively flat-lying basalt of the Cambrian Kalkarindji Igneous Province and Phanerozoic strata of the Georgina Basin (Walter *et al*. 1995; Glass 2002; Glass & Phillips 2006).

## 3 MATERIALS AND METHODS

### 3.1 MT infill survey

**Figure 4.**
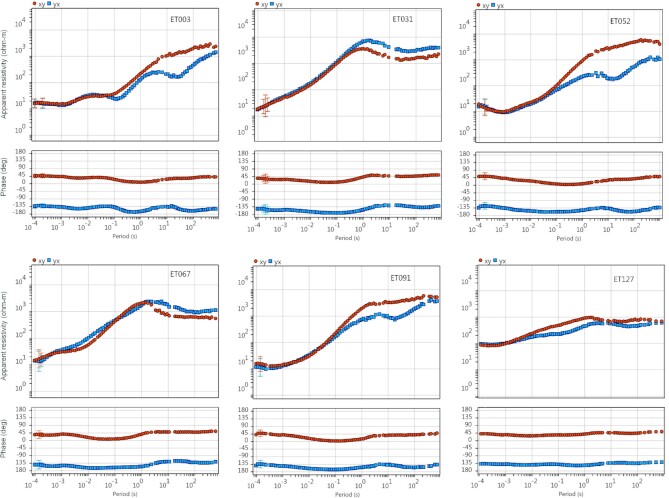
Plots showing processed MT data (apparent resistivity and phase) at six sites spreading across the survey sites that represent the data quality of the whole survey. Note that noisy data at the AMT and BBMT dead bands were masked out.

In 2019 July and August, BBMT and AMT data were acquired at 131 stations with site spacings of approximately 2–10 km (Fig. 2). We designed the MT survey to include three closer spaced transects perpendicular to the geological strike, referred to as the western, central and eastern transects, respectively. These stations were supplemented by a ∼10 km spaced array between transects. Additional data were acquired at proposed drill sites for the purpose of cover thickness estimation. Generally, impedance data were of good quality over periods of 0.0001–1000 s, except data in the dead bands (0.001–0.002 s for AMT and ∼10–20 s for BBMT), in which natural signals are typically weak. The vertical magnetic data were of poor quality and were therefore not used for data inversion. Details of data acquisition and processing can be found in the survey logistics report as part of the data release (Jiang & Duan 2019). Fig. 4 shows the processed data at six sites across the survey region.

### 3.2 Data analysis

The penetration depth of an MT signal represents the exponential decay of the electromagnetic wave amplitude with depth and depends on local resistivity and signal frequency/period. Signals at a given period penetrate deeper in resistive earth material than that in conductive material. It can be estimated using the Niblett–Bostick depth approximation (Niblett & Sayn-Wittgenstein 1960; Bostick 1977; Jones 1983):

(1) \begin{eqnarray*} h = \sqrt {\frac{{\rho \left( T \right)T}}{{2\pi {\mu _0}}}} , \end{eqnarray*}

where *h* is the ‘penetration depth’ in a half-space medium of resistivity equal to the apparent resistivity (*ρ* in Ω·m) at that particular period (*T* in seconds) and μ_0_ is the magnetic permeability value in a vacuum. Note that this penetration depth is for an attenuation factor of approximately 1/2 instead of the more usual skin depth attenuation of 1/*e*.

The definition of a penetration depth is based on the assumption of a homogeneous and isotropic Earth, which means it depends on an averaged bulk resistivity of the subsurface and gives an approximation of resistivity–depth distribution. In practice, the effective depth of investigation is approximately one third of the skin depth, because the sensitivity of impedance *Z* to the conductivity perturbation in the subsurface reduces over depth (Oldenburg 1979). We calculated the penetration depths using the determinant value of the impedance tensor:

(2) \begin{eqnarray*} {Z_{\mathrm{ det}}} = \sqrt {\left| {{{\rm{z}}_{{\rm{xx}}}}{{\rm{z}}_{{\rm{yy}}}} - {{\rm{z}}_{{\rm{xy}}}}{\rm{ }}{{\rm{z}}_{{\rm{yx}}}}} \right|} \end{eqnarray*}

**Figure 5.**
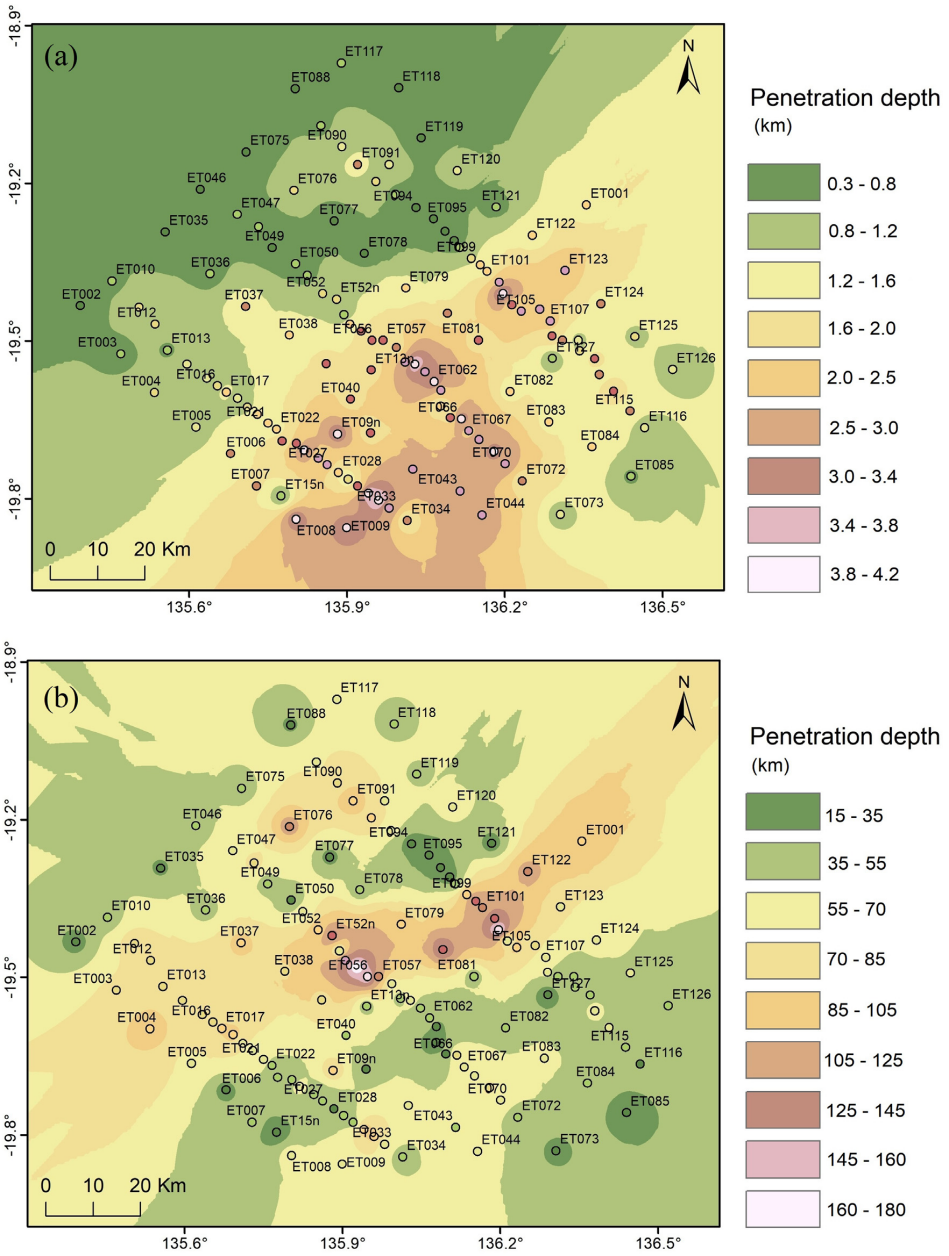
Penetration depth maps at periods of (a) 0.1 s and (b) 100 s, spatially interpolated using the Inverse Distance Weighted (IDW) method. Penetration depths are calculated using the determinant value of the impedance tensor.

As shown in Fig. 5, penetration depths at the survey sites vary significantly across the region, suggesting a complex resistivity structure in the subsurface. At a period of 0.1 s, shallower depths in the northwest reflect thicker conductive cover, indicating the presence of the Georgina Basin, Kalkarindji Province, and likely South Nicholson Basin. Greater penetration depths in the southeast area reflect a thinner cover overlying the resistive basement, indicating the absence of South Nicholson Basin, except at sites ET073, ET085, ET116, ET125 and ET126. At a period of 100 s, penetration depths suggest possible conductive zones at sites ET077, ET095 and ET121; at sites ET006, ET15n, ET028, ET09n, ET066 and ET127, which form a southwest–northeast corridor. This corridor corresponds to the conductivity anomaly mapped in the AusLAMP model and further characterized in 3-D inversion of the infill survey data.

Dimensionality analyses of the MT responses provide qualitative insights into the complexity of the subsurface resistivity structure. We undertook dimensionality analyses using the phase tensor (Caldwell *et al*. 2004; Booker 2014). The phase tensor, defined as the ratio of the real and imaginary parts of the impedance tensor, is formulated to filter local distortion and to derive the underlying regional geoelectric structure (Caldwell *et al*. 2004). It can be graphically depicted as an ellipse. The skew angle measures the degree of asymmetry of the phase tensor, and is an indication of the three-dimensionality of the subsurface. Low ellipticity (circular) and small skew angles (<3°, suggested by Caldwell *et al*. 2004) indicate 1-D or 2-D geoelectric structures, whereas large skew angles indicate 3-D geoelectric structures.

**Figure 6.**
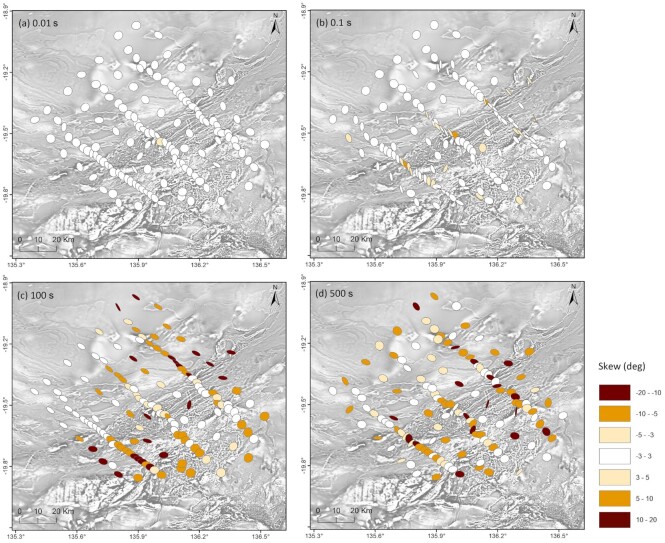
Phase tensor ellipse maps plotted for each sites at periods of about (a) 0.01 s, (b) 0.1 s, (c) 100 s and (d) 500 s, corresponding to the top few hundred metres to mid–lower crustal depths, approximately. Phase tensor ellipses are coloured by skew value indicating dimensionality, with white indicating a skew angle within the threshold for 2-D (±3°), yellow and brown indicating a skew angle out of the threshold. The background map is the first vertical derivative of the total magnetic intensity anomaly map of Australia, 6th Edition (Nakamura & Milligan 2015).

Phase tensor maps are shown in Fig. 6, in which the phase tensor ellipses are coloured by their skew angles. At a short period of 0.01 s, the ellipses at most sites are approximately circular, indicating a dominantly 1-D shallow response. This reflects the flat-lying sedimentary cover sequences. At a period of 0.1 s, the ellipses located in a northeast-trending corridor that is characterized by a linear magnetic grain, have increased ellipticity. This reflects a geoelectric structure change along this corridor in the subsurface. Skew angle at most sites are still within recommended threshold of ± 3° or ± 5° for real data sets, indicating a mixture of 1-D/2-D responses. At longer period of 100 and 500 s, the ellipses are polarized in variable directions, with skew values at some sites outside the range of ± 3°, indicating that the structure is a mixture of 2-D/3-D responses. At longer periods of 100 and 500 s, the ellipses are polarized in variable directions, with skew values at some sites outside the range of ± 3°, indicating that the structure is a mixture of 2-D/3-D responses.

**Figure 7.**
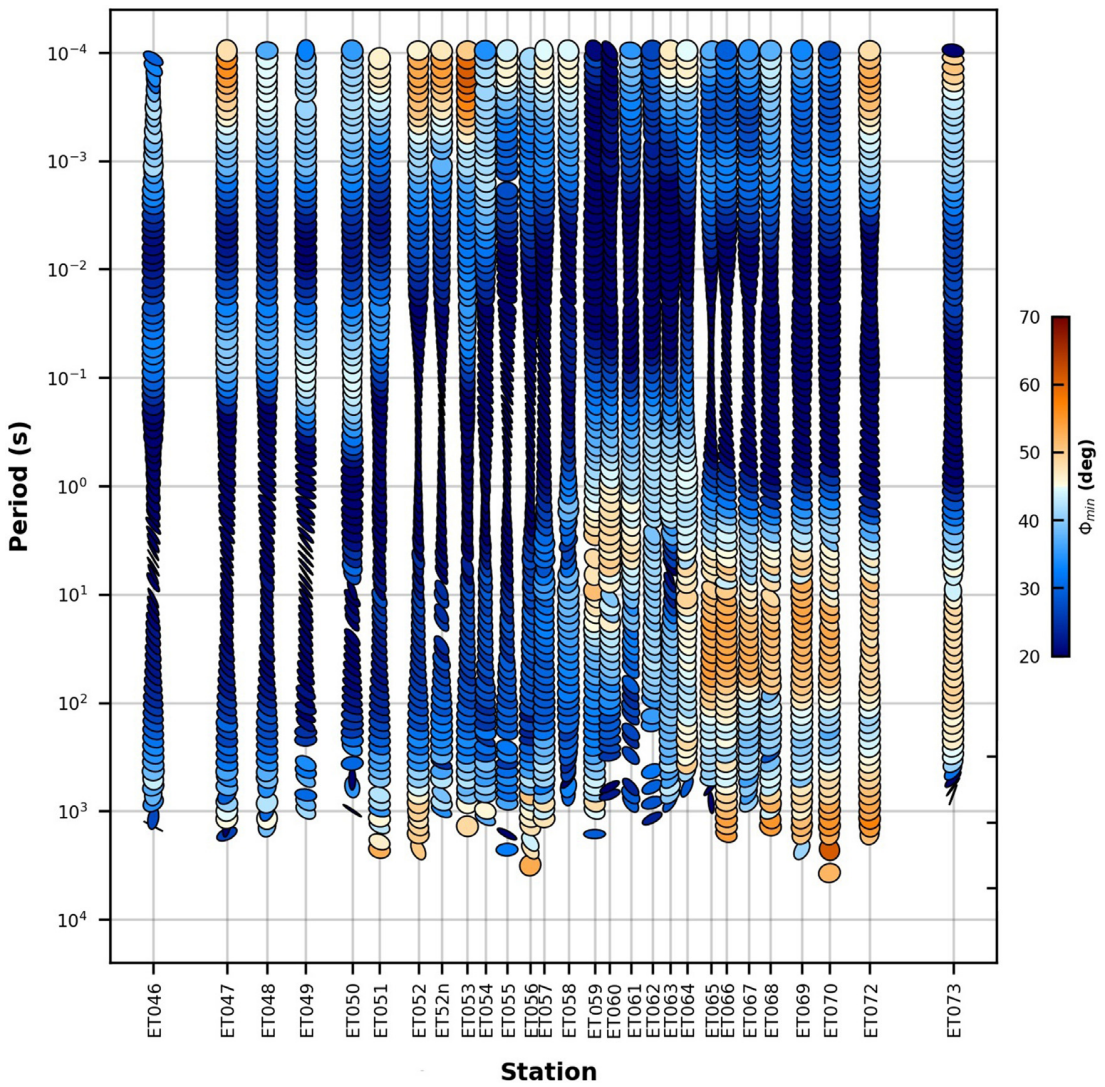
Phase tensors coloured by minimum phase along the central transect. The ellipses are plotted so that the horizontal axis represents the MT sites along the transect and the vertical axis represents period in seconds on a log scale.

Fig. 7 shows the phase tensor pseudo-section coloured by minimum phase values along the central transect. Observed phase values greater than 45° at period ranges of 10–1000 s indicate a transition from a resistive to a conductive zone in the southeast. Thus, the broadband data reveal the presence of a conductive zone at mid–lower crustal levels. This conductive zone is located within the southwest–northeast corridor as shown in Fig. 5(b).

### 3.3 3-D data inversion

In practice, 3-D inversion is often used to interpret complex 3-D subsurface structures (Tietze & Ritter 2013). Dimensionality analysis shows the influence of 3-D structures reflected in the MT responses in the longer period data (>100 s) at some sites (Figs 6c and d). Therefore, 3-D data inversion was undertaken using the 3-D ModEM code (Egbert & Kelbert 2012; Kelbert *et al*. 2014). The inversion algorithms implemented in ModEM include nonlinear conjugate gradients (NLCG, e.g. Rodi & Mackie 2001), data space conjugate gradients (DCG; Siripunvaraporn & Egbert 2007) and the multitransmitter hybrid CG-Occam scheme of Egbert (2012). We applied the standard minimum-structure NLCG algorithm. To improve computational efficiency, the NW–SE profiles were rotated 45° clockwise to align with the N–E model grid. Data were then rotated 45° counterclockwise to keep the same geological strike angle with reference to the source field polarization directions. This manipulation significantly reduced the number of cells and computational resources in our inversion.

**Figure 8.**
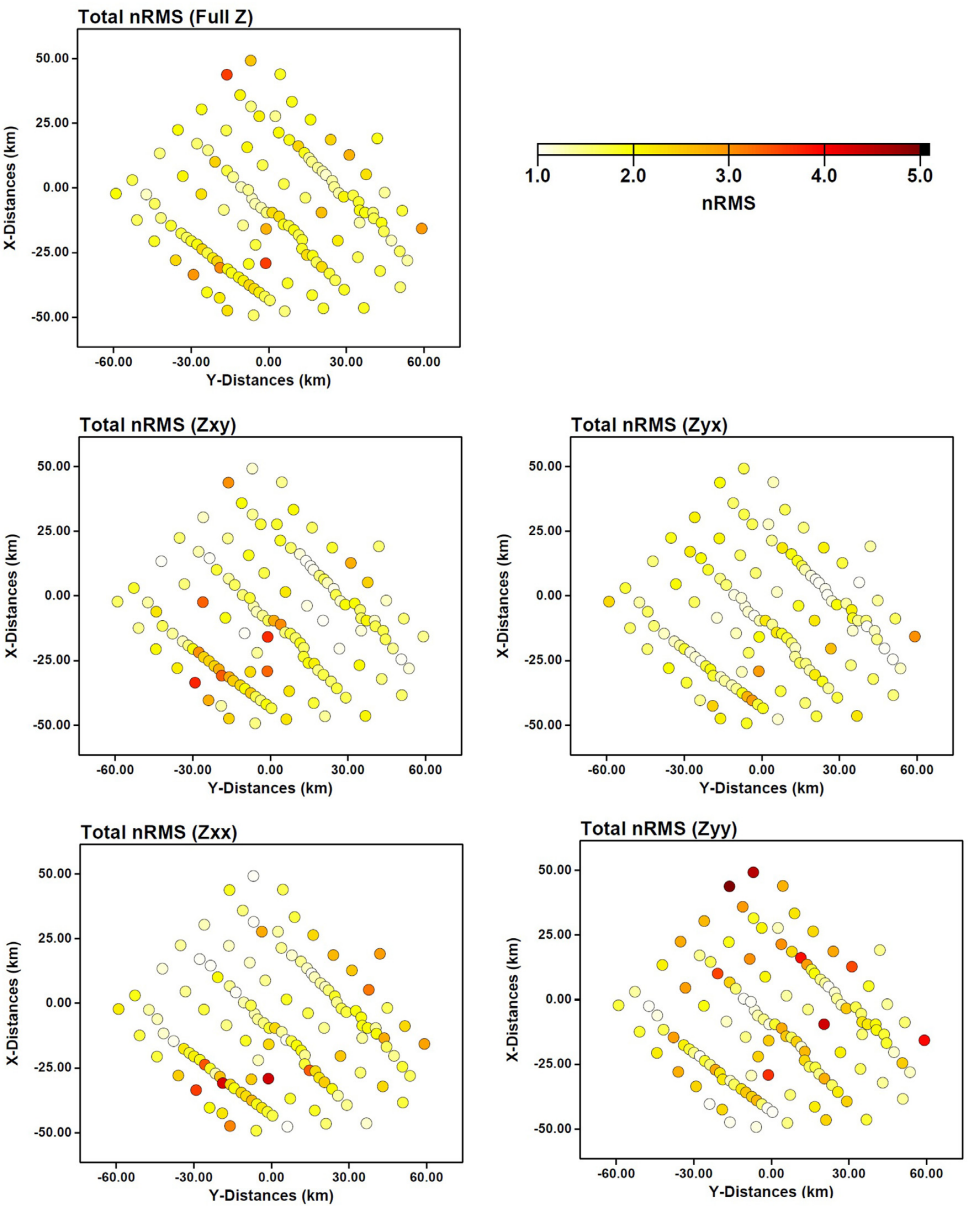
Overall value of noise-normalized RMS misfit across all the survey sites, plotted for the full impedance tensor and four individual components.

We systematically assessed the robustness of major features in the models, by testing starting models and smoothing factors, and using different data subsets and discretization resolutions. The final model consisted of, horizontally, 180 × 140 cells with size of 800 m and 11 padding cells around the perimeter. The size of the padding cells increased by a factor of 1.4. In the vertical direction, the first layer thickness was set as 10 m, and subsequent layer thickness was increased by a factor of 1.1 until the depth of the model reached 855 km (i.e. 2 times greater than the maximum skin depth). Data at 47 periods in the range of 0.0001–1000 s were inverted with error floors of 5 per cent of SQRT(*Z_xy_Z_yx_*) (where *Z_xy_* and *Z_yx_* are the two off-diagonal impedance tensor components) applied to each impedance tensor component. To smooth the model, a covariance value of 0.2 was applied twice in all directions across the model cells. The covariance factor was increased to 0.3 after 108 iterations to smooth the model further. These values were chosen following sensitivity tests of multiple covariance factors. Our preferred 3-D model converged to a noise-normalized root-mean-square (RMS) misfit value of 1.6 after 196 iterations starting from a uniform 100 Ω·m half-space, which is considered to be a reasonably good fit to the data. Fig. 8 gives the distribution of total RMS across the survey sites, indicating an overall good data fit with poor data fit at a few discrete sites. Also shown, data fit varies for differnet impedence tensor components due to sensitivity variations to subsurface structures.

**Figure 9.**
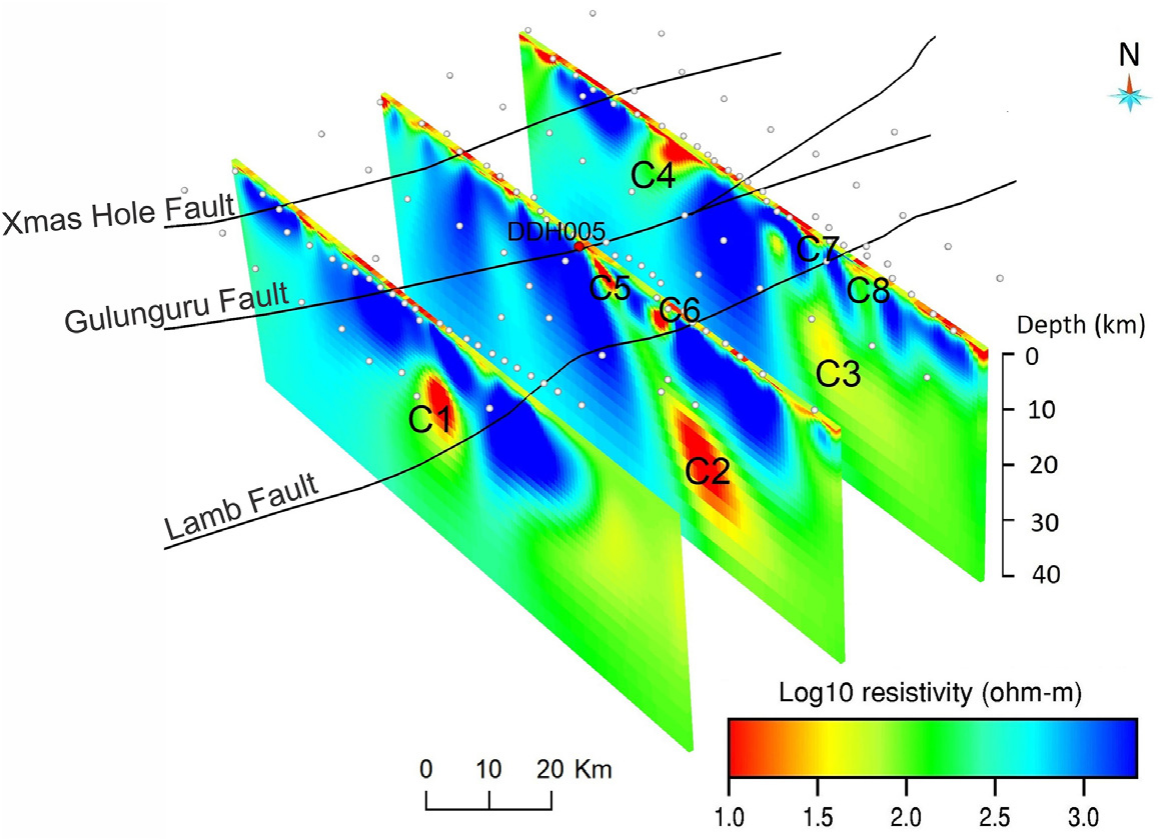
Vertical sections down to 40 km depth along the three denser transects, showing conductors extending from the lower crust to the near-surface. No vertical exaggeration. Black lines = major faults (Clark et al 2021); white dots = MT stations; red dot = location of drillhole DDH005; C1 = conductor 1; C2 = conductor 2; C3 = conductor 3; C4 = conductor 4; C5 = conductor 5; C6 = conductor 6; C7 = conductor 7 and C8 = conductor 8.

**Figure 10.**
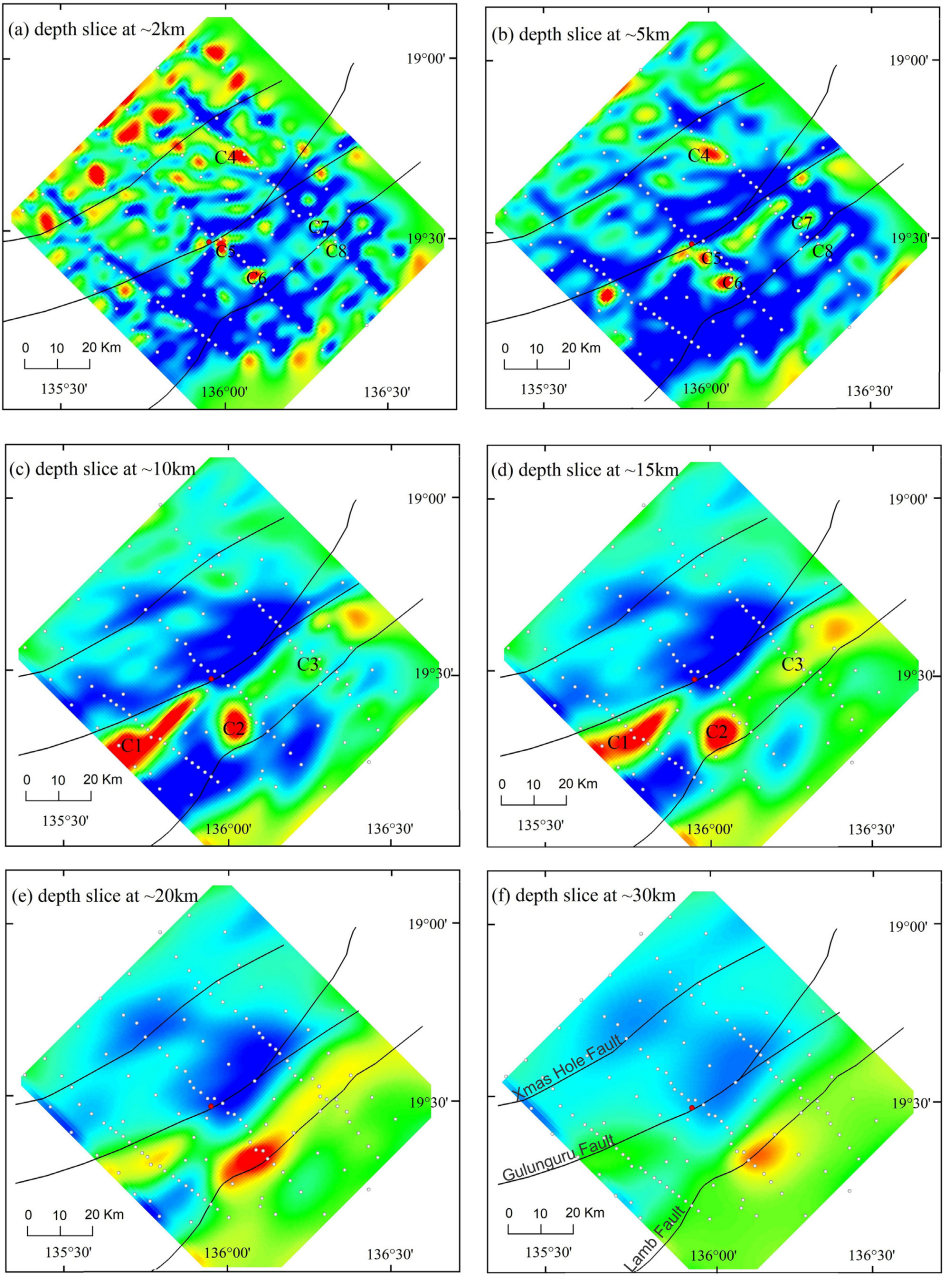
3-D resistivity model depth slices at approximately 2, 5, 10, 15, 20 and 30 km. Black lines = major faults; white dots = MT stations; red dot = location of drillhole DDH005; C1 = conductor 1; C2 = conductor 2; C3 = conductor 3; C4 = conductor 4; C5 = conductor 5; C6 = conductor 6; C7 = conductor 7 and C8 = conductor 8. The colour scale is the same as that in Fig. 9.

**Figure 11.**
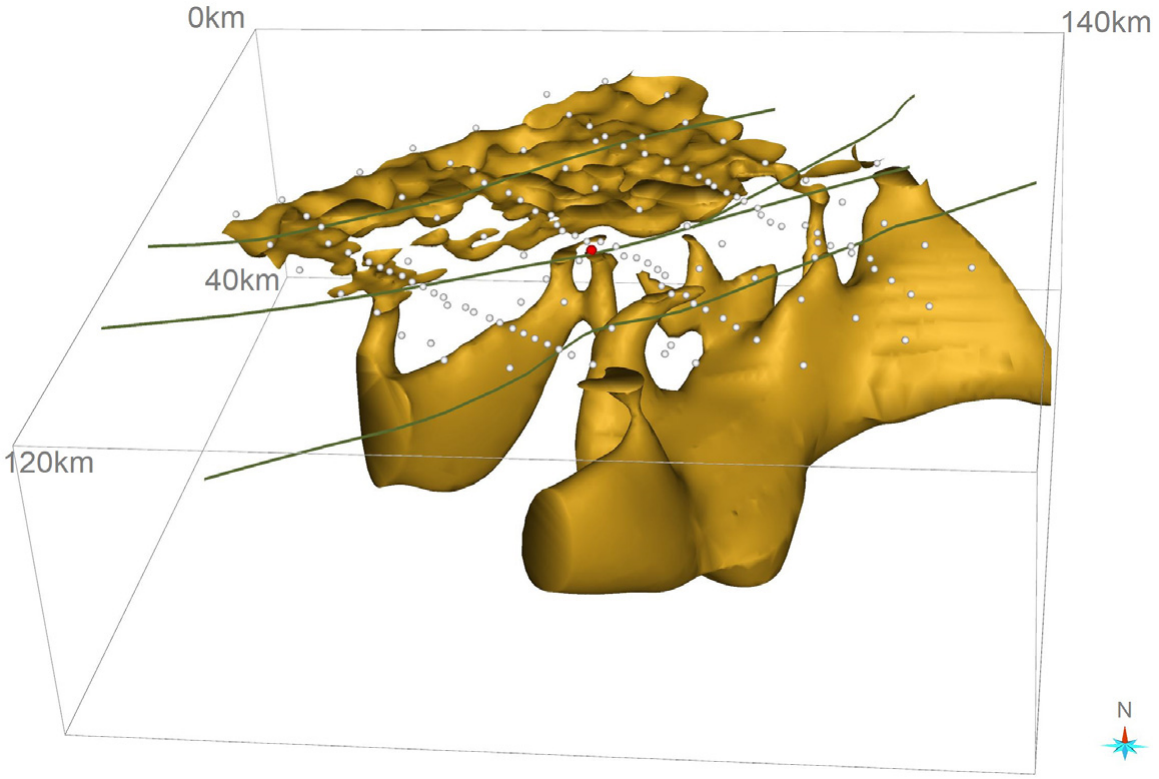
3-D view of the model to a depth of 40 km, showing conductors with resistivity of less than 100 Ω·m. Green lines = major faults (see Fig. 9); white dots = MT stations and red dot = location of drillhole DDH005.

First, we show 3-D model sections along the eastern, central and western transects (Fig. 9). They reveal prominent crustal-scale conductors C1, C2 and C3 in the mid-lower crust, dipping to the southeast. Conductors C2 and C3 extend to the near-surface through conductive pathways (C5 and C6). At the surface, the location of C5 coincides with the Gulunguru Fault and a legacy drillhole (DDH005). Another conductor C4 is observed in the upper crust and is confined to a depth of ∼20 km. Depth slices (Fig. 10) from approximately 2 km to 30 km show that the resistivity contrasts broadly coincide with the major structures. The conductors become fuzzy at a depth of 30 km, indicating the depth resolution the BBMT data can approximately resolve. A 3-D view (Fig. 11) of the conductors (< 100 Ω·m) shows conductors C2 and C3 actually form one conductive body. Also shown is the interconnection of C1 and C2. All these conductors extend to the near-surface via multiple pathways. Interpretation of these conductors is given in Section 4.

### 3.4 probabilistic inversion of AMT data

We applied a probabilistic approach to invert AMT data using our newly developed rj-McMCMT code (https://github.com/GeoscienceAustralia/rjmcmcmt). It is built upon an open-source library called rj-McMC (Hawkins 2013). The algorithm uses trans-dimensional Markov chain Monte Carlo techniques to solve for a probabilistic resistivity-depth model. The trans-dimensional aspect of the algorithm allows the number of layers in the resistivity model to be an unknown. This feature means users do not need to pre-select the model layer structure. This is especially appealing when inverting geophysical data collected in greenfield regions where prior knowledge of the geology may not be adequate and pre-selection of layers can bias results.

The inversion of each station employs multiple Markov chains in parallel to generate an ensemble of millions of resistivity models that adequately fit the data given the assigned noise levels. Once the ensemble of models is generated, its statistics are derived from the posterior probability distribution (PPD) of the resistivity at any particular depth, as well as the number of layers and the depths of the interfaces. This probabilistic approach gives a thorough exploration of the model space and a more robust estimation of uncertainty than deterministic methods allow. The inversion results are driven by data and relatively uninformed uniform priors; therefore, they are global and robust (Chen *et al*. 2012). The downside is that the sampling process is more computationally expensive and is only applicable to 1-D, or 2-D with fast forward modelling. However, recent research effort has developed fast solutions for 3-D forward problem, which is to construct an approximation or surrogate model to avoid running a full forward model (e.g. Manassero *et al*. 2021). For details of the algorithm, refer to Brodie & Jiang (2018).

**Figure 12.**
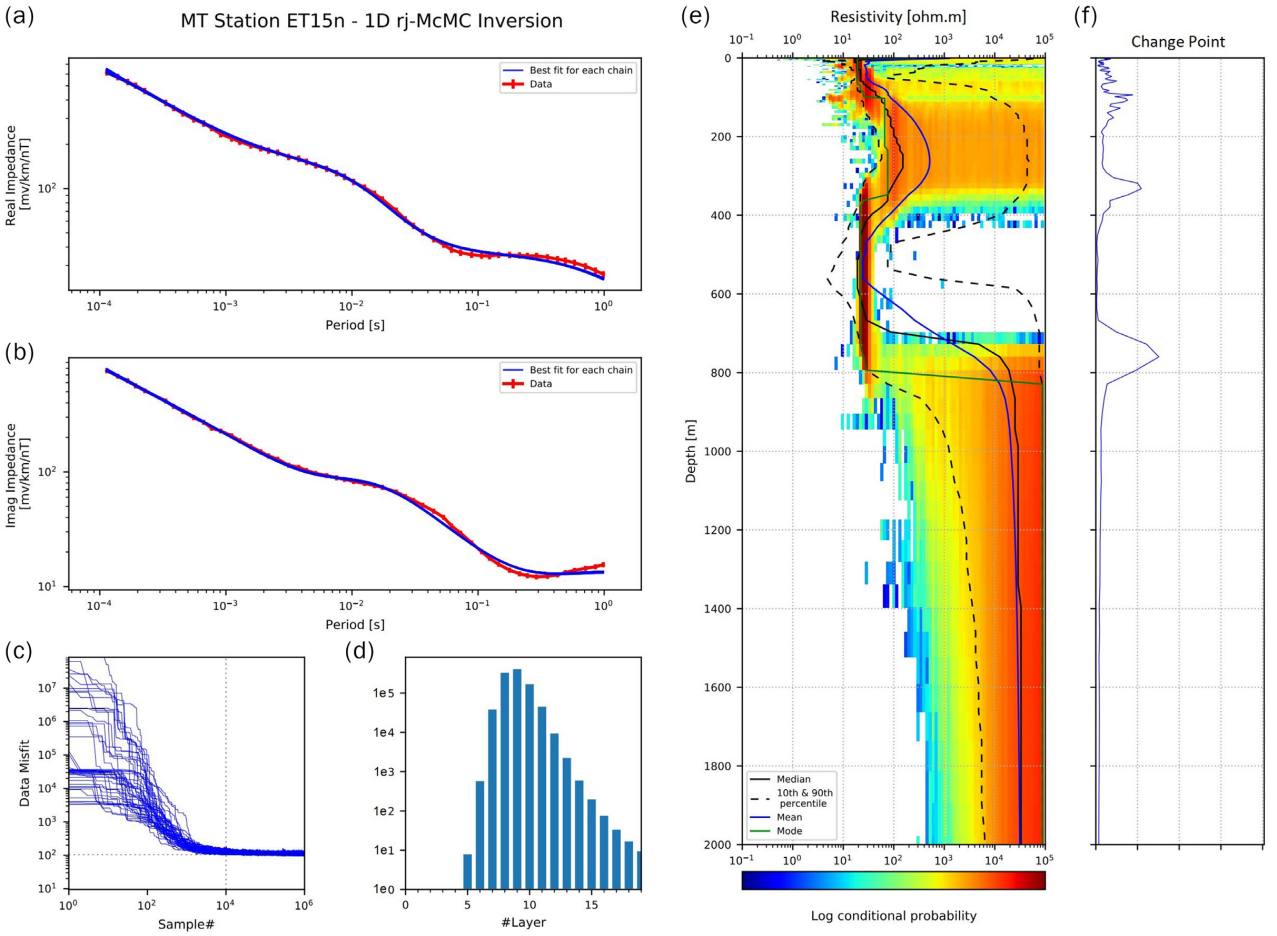
Plot summarizing the results of the rj-McMCMT inversion: (a) and (b) real and imaginary impedances and error bars (red) and the best-fitting model from each Markov chain (blue); (c) data misfit convergence history for each Markov chain; (d) histogram of the number of model layers; (e) the summary median, 10th and 90th percentile, mean and mode models overlying the pseudo-coloured shaded image of the 2-D log-PPD histogram and (f) the change-point histogram showing the probability of where layer interfaces occur.

We inverted the determinant of the impedance tensor at each site, using 32 Markov chains with each sampling 1 million models. Fig. 12 shows an example of model results. The number of samples (models) for the burn-in period is specified as 10 000, which allows the data misfit to converge to an acceptable level before any samples are accepted into the ensemble. A discretized 2-D PPD histogram (Fig. 12e) is built up by incrementing all resistivity-bin counts, as well as a 1-D depth interface change-point histogram (Fig. 12f), which describes the probability of a resistivity layer interface occurring at a particular depth bin. The 2-D PPD histogram with pseudo-coloured shading indicates higher probability in warmer colours and low probability in colder colours. Also shown are the summary models, including median, 10th and 90th percentile, mean and mode models.

The probabilistic modelling approach gives pronounced layer boundaries that facilitate a more straightforward interpretation of resistivity structure. The estimation of cover thickness at each site was produced by utilizing the resistivity contrast between the overlying sedimentary basins and the basement. The 1-D change-point histogram (Fig. 12f) can be interrogated to assess the probability of a peak falling at a particular depth and in some cases can be used to quantify layer boundary uncertainty. In this study, the full width at half maximum (FWHM) is used to estimate the uncertainty of a layer boundary interface. The FWHM is calculated by finding the width in metres of the peak at half of its maximum probability. Estimated cover thickness and uncertainty at the proposed drill sites are summarized in Table 1. Interpretation of these results is given in Section 4.

**Table 1.** Cover thickness estimates from the rjMcMCMT 1-D inversion results at the proposed drill sites, along with uncertainty quantified by FWHM in metres.

Site ID	Latitude	Longitude	Elevation (m)	Cover thickness estimate (m)	FWHM (m)
ET001	−19.2411	136.3554	224	66.5	8.50
ET10n	−19.5727	136.1261	221	197.3	17.17
ET11n	−19.181	135.9084	212	279.5	48.69
ET12n	−19.7856	136.1155	227	127.7	11.12
ET13n	−19.5556	135.9459	222	1337.9	660.48
ET15n	−19.7952	135.7746	232	760.0	132.41
ET02n	−19.5666	135.6911	222	986.7	171.89
ET52n	−19.4205	135.88	220	1459.5	417.93
ET92n	−19.1642	135.9798	219	224.9	39.17
ET127	−19.5338	136.2905	235	318.4	122.76

## 4 INTERPRETATION AND DISCUSSION

This section concentrates on the interpretation of results from the infill MT survey, which provide relatively high-resolution imaging of the crustal-scale structures and the near-surface features. We also incorporate seismic reflection data collected along line 19GA-B1 to provide further constraints on the structural architecture of the area.

### 4.1 Sedimentary sequences in the east tennant region

**Figure 13.**
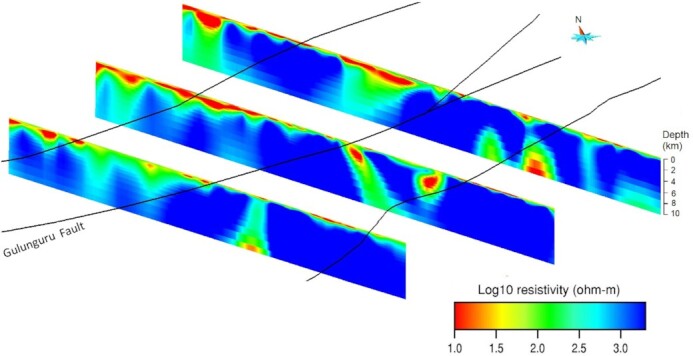
Three planar vertical surfaces to a depth of 10 000 m extracted from the 3-D resistivity model along the western (bottom), central (middle) and eastern (top) transects, aligned to their position relative to the Gulunguru Fault. No vertical exaggeration.

Three planar vertical sections to a depth of 10 000 m were extracted from the 3-D resistivity model along the denser transacts (Fig. 13). Conductive materials are observed to a greater depth (a few hundred to ∼2000 m) to the northwest of the Gulunguru Fault, possibly representing the black shales within the basin sequences, for example, the Mullera Formation of the South Nicholson Basin. The recessive Mullera Formation contains organic-rich shale and minor ironstone, which contribute to enhanced conductivities. Its thickness is estimated to be greater than 1100 m in the Northern Territory (Carter & Zimmerman 1960). Based on our results, we suggest that the Mullera Formation could be present at a depth of a few hundred metres and reaching substantial thickness northwest of the Gulunguru Fault in the East Tennant region. Results from 3-D models also indicate that the Palaeo- and Mesoproterozic basin sequence is fault bounded by the Gulunguru Fault, suggesting a thinner cover of only Georgina Basin and Kalkarindji Province (a few hundred metres) in the hangingwall of the Gulunguru Fault, resting upon highly resistive basement (>1000 Ω·m).

The 1-D model with a finer discretization, for example, at site 15n (Fig. 12), shows a near-surface conductive layer of approximately 50 m (<20 Ω·m), interpreted to be the weathered top of the Georgina Basin. Beneath is a moderately resistive layer (a few hundred Ω·m), consistent with a mixture of sedimentary rocks of Georgina Basin and the volcanic Helen Springs Group of the Kalkarindji Igneous Province (Glass 2002; Glass & Phillips 2006). The boundary between these two layers, however, is not discernible. This is due to the diffusive nature of electromagnetic waves, where sharp boundaries and thin layers are not clearly defined (i.e. are smeared out) in the models. At greater depths (∼350–800 m), conductive materials are observed overlying the highly resistive basement.

Cover thickness estimates at proposed drill sites have assisted with stratigraphic drill targeting. For example, as given in Table 1, cover thickness estimate at ET92n confirms a newly recognized basement high, which is at a drillable depth (∼220 m). At site ET02n and ET52n, the top of basement is likely as deep as ∼1000 m due to the presence of South Nicolson Basin. Therefore, this site was not drilled. At site ET15n, model result suggests the basement is unlikely to be reached until ∼760 m. Therefore, this site was moved to ∼12 km northeast. All the final drill sites are selected to the southeast of Gulunguru Fault, except site ET92n.

### 4.2 Crustal structural architecture

The 3-D resistivity model derived from infill MT survey has resolved a number of structures within the resistive host, including two prominent crustal-scale conductors [C1, C2 and C3 (form one conductor)], whose combined responses result in the large-scale conductivity anomaly mapped in the AusLAMP model (Duan 2019; Duan *et al*. 2020). Fig. 11 shows the two conductors are interconnected with multiple conductive pathways extending to the near-surface where the major faults are located. Shown in Fig. 10, conductor C5 coincides with the position of Gulunguru Fault on the surface and is connected to the deeper conductor C2. This observation characterizes the Gulunguru Fault as a southeast-dipping structure extending to a depth of approximately 40 km. Likewise, conductors C8 and C3 characterize the Lamb Fault as a southeast-dipping structure to a depth of approximately 30 km. On the contrast, the Xmas Hole Fault located to the northwest seems not affiliated with a conductivity anomaly.

We interpret the coincidence of mapped deep-penetrating faults and high conductivities to suggest that these faults potentially acted as pathways for transporting metalliferous fluids to the upper crust to form mineral deposits, as recognized by previous studies (e.g. Drummond *et al*. 2000; Willman *et al*. 2010; Johnson *et al*. 2013). Conductive crustal pathways may mark the prevailing alteration from mineralizing fluid migration through these structural zones, and often show a spatial correlation with mineral occurrences in the upper crust (e.g. Heinson *et al*. 2006; Dennis *et al*. 2011; Robertson *et al*. 2015). Similar structural controls were observed in mineralized terranes and were associated with IOCG discoveries (Skirrow *et al*. 2018). Examples include Olympic Dam in South Australia (Heinson *et al*. 2006; Heinson *et al*. 2018) and Mount Isa in Queensland, Australia (Lilley *et al*. 2003; Wang *et al*. 2018; Jiang *et al*. 2019). The implications of results from this study on mineral systems are further discussed in section 5.

Elsewhere, certain types of mineral deposits show a close spatial relationship with craton and palaeocraton margins, for example, lode gold deposits in the Yilgarn Block, Western Australia (Groves *et al*. 1989), and Ni–Cu–PGE sulphide deposits in North Atlantic, Southern Africa, Siberia, China and Australia (Begg *et al*. 2010). The majority of these types of deposits appear to form during periods when collisions, basin inversions, and regional contraction are dominant (Begg *et al*. 2010).

### 4.3 Integration with seismic reflection data

**Figure 14.**
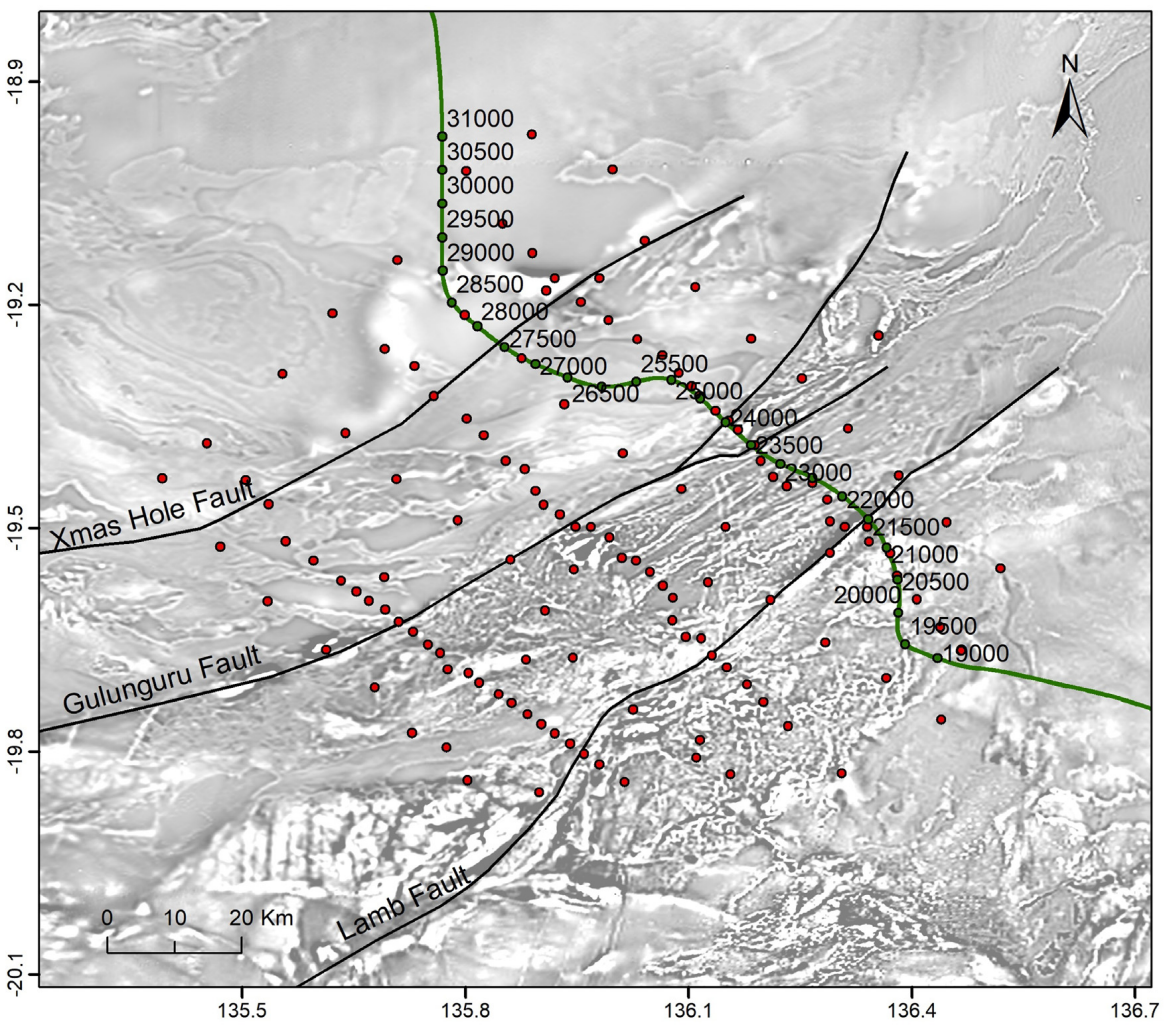
Green dots show the location of a segment of deep seismic reflection line 19GA-B1 (Southby *et al*. 2021) that transects the north-eastern part of the East Tennant region. Red dots are the MT stations (refer to Fig. 2 for the site names). The background map is the first vertical derivative of the total magnetic intensity anomaly map of Australia, 6th Edition (Nakamura & Milligan 2015).

**Figure 15.**
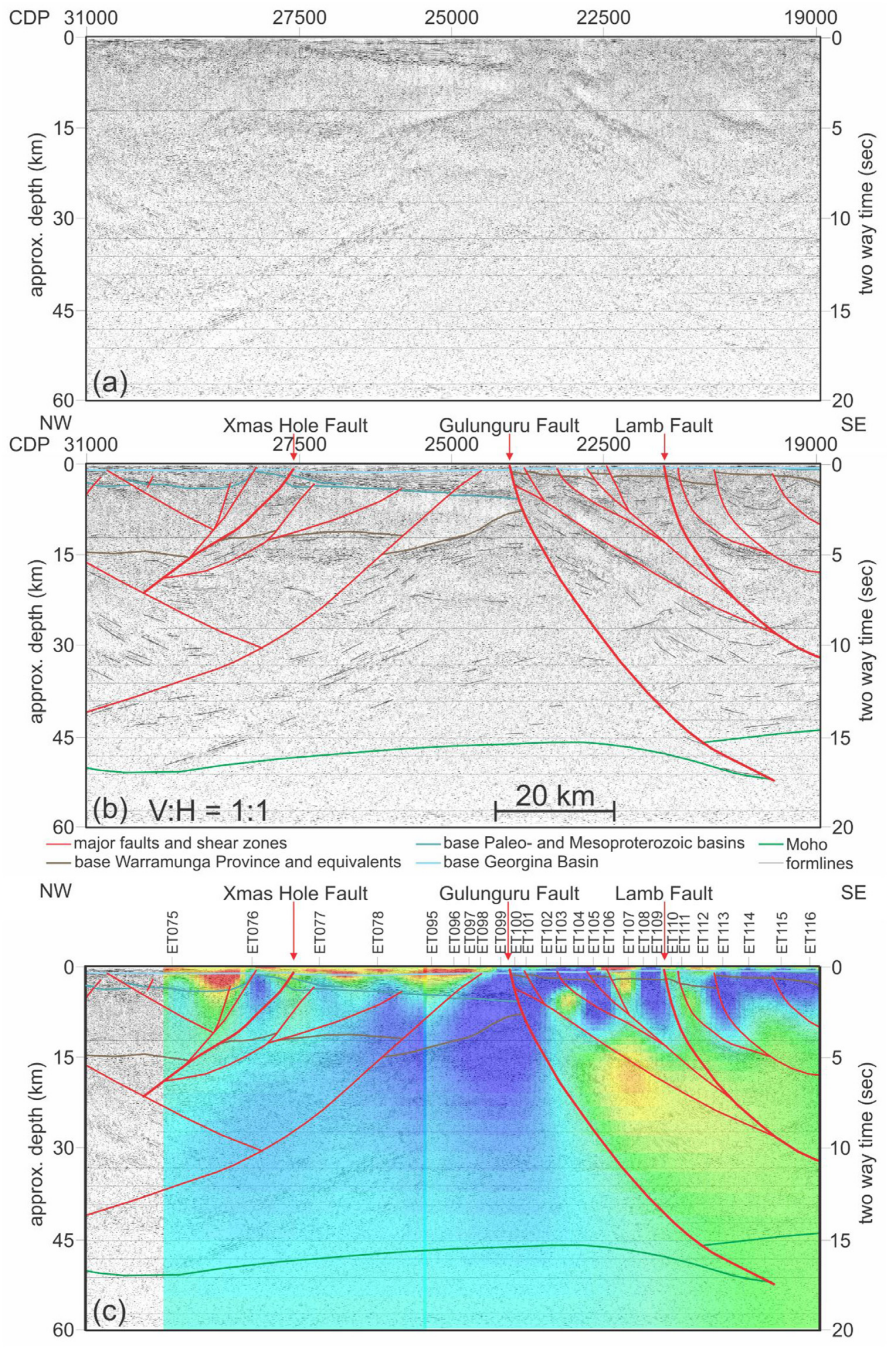
(a) A segment of deep seismic reflection line 19GA-B1 (Southby et al., in preparation) that transects the north-eastern part of the East Tennant region, at a scale of V:H = 1:1. (b) Deep seismic reflection profile with the geological interpretation overlain. (c) The 3-D resistivity model sections in line with the seismic line with the seismic image and geological interpretation overlain. The resistivity model has the same colour scale as that in Fig. 9.

To complement the MT interpretation, we utilize a c. 120 km long segment of deep seismic reflection line 19GA-B1. This segment transects the eastern part of the East Tennant region (Fig. 14) and provides insight into the regional geology and further constraints on the structural architecture of the area (Fig. 15). Line 19GA-B1 has been acquired as part of the EFTF Barkly deep seismic reflection survey, and for technical details and an interpretation of the entire line we refer to Southby *et al*. (2021).

Shown in Figs 15(a) and (b), the seismic data reveal the Gulunguru Fault as an SSE-dipping major crustal boundary that offsets the Moho, with thinner crust (∼45 km thickness) observed to the southeast. In its hanging wall, the structural architecture is dominated by a set of (apparently) SE-dipping faults with listric and/or planar geometry, some of which are interconnected at depth. The Lamb Fault is one of these structures and extends to a depth > 30 km. This interpretation is consistent with the constraints provided by the MT data, where major structures such as the Gulunguru Fault and the Lamb Fault are not confined to upper crustal levels but continue into the middle to lower crust (Fig. 15c).

In the hangingwall of the Gulunguru Fault, metamorphic and magmatic basement rocks of the Warramanga Province are characterized by a relatively bland seismic character and are interpreted to be a few km thick. The remainder of the crust is formed by strongly to moderately reflective rocks that are interpreted as the crystalline basement substrate to the Waramanga Province, likely of late Archean to earliest Palaeoproterozoic age (Champion 2013).

To the northwest of the Gulunguru Fault, the structural architecture is dominated by northerly dips. A major structure in this area is the Xmas Hole Fault that is truncated in the middle crust. A seismically bland package of rocks overlying the Archean crystalline basement and resembling the character of the Warramunga Province is significantly thicker than further to the south, reaching > 10 km around common depth point (CDP) 31 000. The package is overlain by a basin sequence, the upper part of which is intersected by drillholes and identified as part of the Mesoproterozoic South Nicholson Basin (Kruse 2003). This Palaeo- and Mesoproterozic basin sequence occurs in two sub-basins that are separated by a basement high in the hangingwall of the Xmas Hole Fault. In the MT data, this basement high is recognized/highlighted as a zone of low conductivity (Fig. 15c). Both sub-basins show some thickening towards the SE, although this is more pronounced for the southern one. The Georgina Basin and Kalkarindji Province overlie all of the area, but seem slightly thicker than to the south of the Gulunguru Fault.

An important observation based on the integration of the MT data with the deep seismic reflection interpretation is that the Gulunguru Fault is bounding two lithospheric blocks with distinctly different resistivities (Fig. 15c). In the northwest, the basement is coherently resistive, with all conductive features confined to upper crustal basins. The conductive basement is entirely confined to the hangingwall of the structure, underpinning the significance of the Gulunguru Fault not only as a mantle-tapping structure, but also as a major crustal boundary separating different lithospheric blocks.

The conductive zones (C2 and C5) revealed in the resistivity model (Figs 9 and 10) approximately coincide with the position of the Gulunguru Fault on the surface. Such conductive zones extend to the lower crust across the Moho and are likely connected to the conductive zone in the upper mantle mapped in the AusLAMP model (Duan 2019; Duan *et al*. 2020). Moho offset at the lower crust marked by the Gulunguru Fault could provide a locus for crustal deformation and the establishment of possible fluid pathways (Wise & Thiel 2020).

Deep seismic reflection and magnetotelluric data provide a rare opportunity to map the crustal architecture and imprints of past geodynamic processes. Similar to our finding, Haugaard *et al*. (2021) have mapped a heterogeneous crustal architecture along the Gold-Endowed Abitibi Greenstone Belt in the Archean Superior Province of Canada. They revealed a major subvertical, low-resistivity and seismically transparent corridor that connects the lower crust with the upper crust, which was interpreted as a deep-rooted extensional fault system that acted as a regional-scale conduit for gold-bearing hydrothermal fluids from a source region in the lower crust to transport to depositional sites in the upper crust. Hill *et al*. (2021) suggest that the preserved low-resistivity source region represents an amalgamation of magmatic-hydrothermal and deformational processes that occurred during episodic evolution in the Archean.

## 5 IMPLICATIONS FOR MINERAL SYSTEMS

We demonstrate an integrated methodology to map multiscale footprints of mineral systems, which can be used to identify new provinces for mineral exploration in covered terranes. The approach and workflow we adopted map to the conceptual framework of mineral systems, and its four main components essential for mineral deposit formation (Wyborn *et al*. 1994; Skirrow *et al*. 2019; Murr *et al*. 2020).

First, the AusLAMP model (Duan 2019; Duan *et al*. 2020) mapped a large-scale conductivity anomaly in the mid–lower crust and upper mantle in the East Tennant region. This zone has higher conductivities than what would be expected for dry, crystalline lithosphere and, therefore may represent a fertilized source region related to mineral system processes.

Second, multiple geophysical data suggest favourable lithospheric architecture for large (crustal)-scale fluid migration. From seismic reflection and potential field data, we interpreted a number of large-scale structures that characterize the east-northeast-trending East Tennant basement high. Passive seismic data mapped an offset of the Moho discontinuity with a deeper Moho in the north relative to the south (Sippl 2016; Gorbatov *et al*. 2020), consistent with reflection seismic constraints showing such an offset along the Gulunguru Fault (Fig. 15b). In addition, passive seismic data revealed this region is transected by a major gradient in the lithosphere–asthenosphere boundary (LAB), with ∼40 km of vertical offset across 150 km of lateral distance (Czarnota *et al*. 2020), which coincides with a north-easting corridor in the middle crust (Hejrani *et al*. 2020, Fig. 16). This lithosphere-scale structural corridor has been refined by results from higher-resolution infill MT survey in this study, which further confirmed with a greater level of detail the existence of a fluid pathway architecture linking conductive zones in the lower crust and upper mantle with (potential) depositional sites in the upper crust.

**Figure 16.**
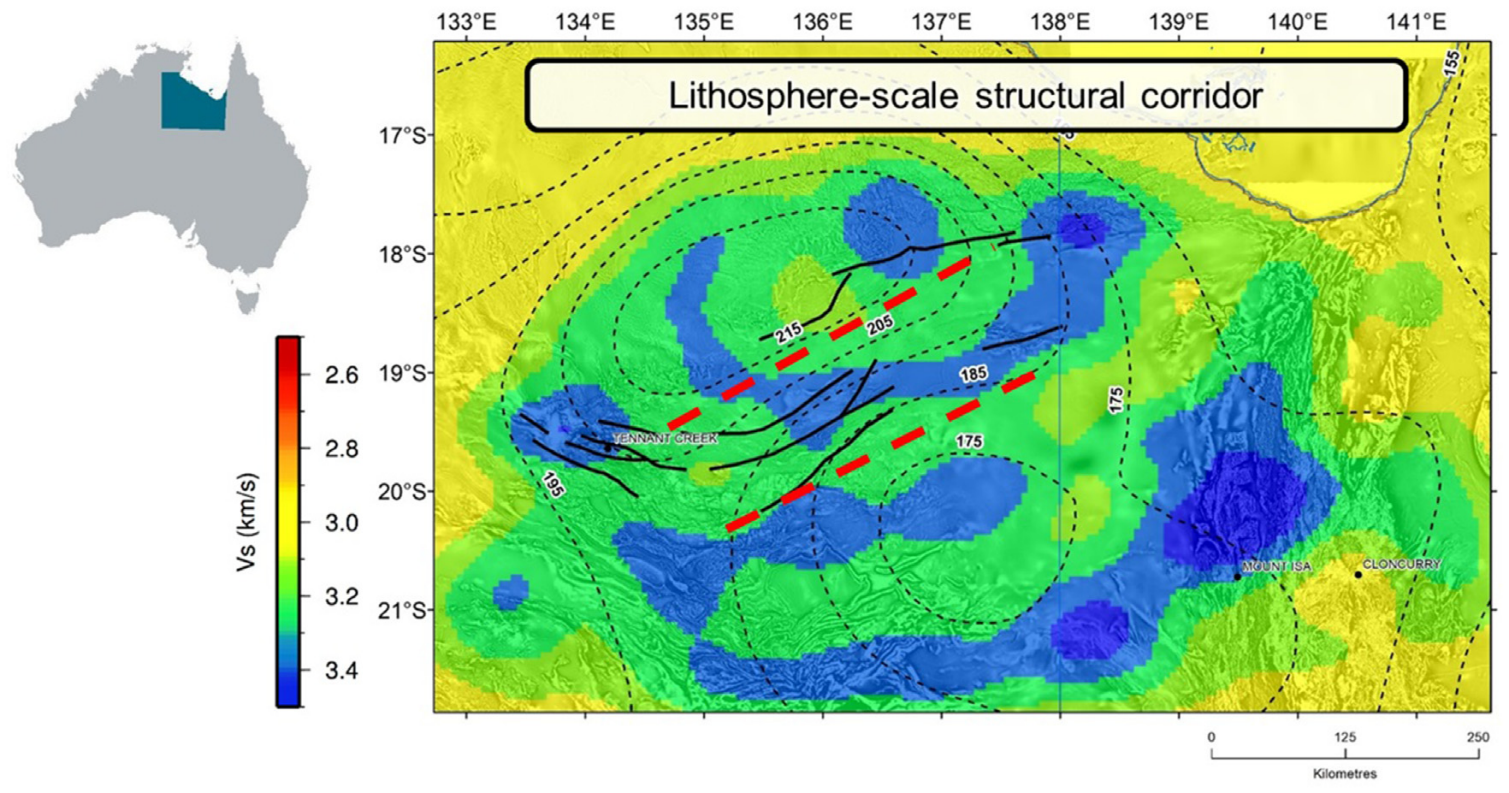
The contour of the LAB (boundary of lithosphere and asthenosphere) overlain on a velocity image at 10 km depth in the area between Tennant Creek and Mount Isa. Combined image from Czarnota *et al*. (2020) and Hejrani *et al*. (2020).

Third, new geochronological data (Cross *et al*. 2020) suggest basement rocks in the East Tennant region have been pervasively affected at ca 1850 Ma by the Tennant Event, that is known as a major tectono-magmatic event in other parts of the Warramunga Province (e.g. Tennant Creek district). It is therefore an event of significant regional extent that could constitute an energy driver component of a mineral system.

Finally, modelled magnetite and haematite proxies from potential field data represent potential locations for iron-oxide alteration related to IOCG-type mineralization throughout the East Tennant region (Goodwin & Skirrow 2019). In particular, drill core sample from a historical drillhole (DDH005 in Figs 9 and 10) shows the presence of schist with pervasive magnetite and haematite alteration (Skirrow *et al*. 2019; Schofield *et al*. 2020). These results suggest that the four essential components for mineral deposit formation coexist in the East Tennant region, which supports the inference from predictive mapping (Murr *et al*. 2020) that the East Tennant basement high is likely to be prospective for IOCG type of deposits.

Results from our infill MT survey have revealed two prominent crustal-scale conductors with multiple conductive pathways extending to the near-surface in coincidence with or in close proximity to mapped major faults, that is, the Lamb Fault and the Gulunguru Fault. Possible sources for enhanced conductivity in the crust include aqueous fluids, partial melts or metallic compounds (Jones 1992; Ferguson *et al*. 1999; Becken *et al*. 2011; Myer *et al*. 2013). However, in tectonically stable lithosphere, we would not expect aqueous fluids or magmatism to exist in the crust, because the porosity and brine conductivity would have to be unreasonably high at mid-crustal depths (Archie 1942; Heinson *et al*. 2005; Storvoll *et al*. 2005), and, because crustal hydrothermal cells involved in the Tennant Event are unlikely be present after 1850 Myr. Rather, the conductivity anomalies may record the activity of fluid hydration during past tectonic events, with the enhanced conductivity being likely related to the remnant (metallic) material deposited when fluids were present during the ‘ancient’ active deformation/oogenesis. Some possible sources include iron sulfides (such as pyrite and pyrrhotite), iron oxides (magnetite) and graphite (e.g. Selway 2014; Robertson *et al*. 2015; Corseri *et al*. 2017). Preliminary petrological studies from a historical drillhole (DDH005) in the East Tennant region indicate the presence of IOCG-type hydrothermal alteration, including magnetite-rich, potassic and later haematite–sericite–chlorite alteration (Skirrow *et al*. 2019; Schofield *et al*. 2020). Therefore, we suggest that there is high potential for IOCG-type deposits to be found in the general vicinity of the major structures, such as those high-grade magnetite-replacement Au–Cu deposits at Tennant Creek.

From an exploration perspective, mapping sedimentary basins and covered near-surface geological features supports the effective search for mineral deposits in the upper crust. Interpretation of the MT data has improved our understanding of the distribution and geometries of sedimentary basins undercover; in some cases, their configurations were previously interpreted based on sparse drillhole and potential field data. Our results have assisted with the planning of a regional drilling program, and helped reduce the uncertainty associated with intersecting targeted stratigraphic units. Geoscience Australia in partnership with MinEx CRC has successfully completed a 10-hole, 4000 m drilling campaign in the East Tennant region, as part of a world-first scientific drilling program, the National Drilling Initiative (NDI). Comprehensive analyses are planned to characteriszethe drill core which will provide insights into the geological evolution and mineral systems potential of the region. The results will also be used to validate the models and improve our geophysical interpretations.

## 6 CONCLUSIONS

This study demonstrates that integration of geophysical data from multiscale surveys is an effective approach to mapping the footprint of mineral systems at a variety of scales. This helps to reduce the search space and, ultimately, to identify associated mineral deposits in covered terranes.

We have used long-period data from the AusLAMP as a first-order reconnaissance survey to resolve large-scale lithospheric architecture for mapping areas of mineral potential in northern Australia. A 3-D resistivity model reveals a broad conductivity anomaly extending from the Tennant region to the Murphy Province in the lower crust and upper mantle, representing a potential fertile source region for mineral systems (Duan 2019; Duan *et al*. 2020). Results from higher-resolution infill magnetotelluric survey reveal two prominent conductors in the resistive host whose combined responses result in the lithospheric-scale conductivity anomaly mapped in the AusLAMP model. Most importantly, conductive structures indicate a ‘favourable’ crustal architecture linking the lower, fertile source regions with potential depositional sites in the upper crust. This assertion is strengthened by the deep seismic reflection data, illustrating that the major faults indeed are deep-penetrating structures that potentially acted as pathways for transporting metalliferous fluids to the upper crust where they could form mineral deposits. Our result and its integration with other data sets support a high prospectivity for major mineral deposits in the East Tennant region.

In addition, cover thickness estimation from high-frequency AMT data has assisted with stratigraphic drill targeting which, in turn, will be used to validate the models and improve our understanding of basement geology, cover sequences and mineral potential.

## Data Availability

AusLAMP data collected in the Tennant Creek-Mt Isa region have been released by Geoscience Australia via http://dx.doi.org/10.11636/134997. BBMT and AMT data from the infill survey in the East Tennant region have been released by Geoscience Australia via http://dx.doi.org/10.26186/5df80d8615367.
